# Odontogenic Myxoma in Children: A Case Report and Literature Review

**DOI:** 10.1155/2016/9017421

**Published:** 2016-03-15

**Authors:** Mariana Dalbo Contrera Toro, Icléia Siqueira Barreto, Eliane Maria Ingrid Amstalden, Carlos Takahiro Chone, Leopoldo Nizam Pfeilsticker

**Affiliations:** ^1^Department of Otorhinolaryngology, Head & Neck Surgery, State University of Campinas (UNICAMP), P.O. Box 6111, 13081-970 Campinas, SP, Brazil; ^2^Department of Ear, Nose, Throat and Head & Neck Surgery, Unicamp, Brazil; ^3^Department of Pathology, Unicamp, Brazil; ^4^ENT, Maxillo Facial, Maxilo Facial ENT Service, Unicamp, Brazil

## Abstract

Benign odontogenic lesions are rare entities but are very important due to their locally aggressive nature. Odontogenic myxoma is even rarer in children than in adults. There is no evidence in the literature in regard to the best treatment approach, in terms of conservative or aggressive surgery, for this type of tumor. This paper reports a case of odontogenic myxoma in a child treated with a compromised approach through bone osteotomies and a review of the literature about this disease, especially in pediatric patients.

## 1. Introduction

Odontogenic myxoma is a rare tumor that occurs in the mesenchymal tissue of the mandible and maxilla and represents 3–6% of all odontogenic tumors. Despite its benign character, it can be locally aggressive and has a recurrence rate of 25% [1]. It is usually diagnosed in the third decade of life and has no gender predilection [2]. In children, it can occur even less frequently than in adults but tends to have the same pathologic and clinical features.

Most patients are asymptomatic at diagnosis and typically only present with image findings of the tumor. However, the tumor can also present as a facial deformity with no associated pain or inflammation [2].

The diagnosis consists of clinical findings along with an image study (radiography, tomography, and MRI) but can only be confirmed by histologic analysis. Radiography usually shows a mandibular or maxillary tumor that is unilocular with trabeculae and that is sometimes associated with tooth displacement. Tomography shows a radiolucent tumor with trabeculation, and although typical radiography can be enough for diagnosis, CT and MRI are essential to surgical planning and the evaluation of the tumor extension [3].

There is no evidence-based consensus in terms of treatment of the odontogenic myxoma. A literature review demonstrated a large spectrum of surgical treatments varying from conservative enucleation of the tumor to aggressive resections such as mandibulectomy and maxillectomy. This study reports a rare case of odontogenic myxoma in a pediatric patient and discusses diagnosis and treatment options according to a literature review.

## 2. Materials and Methods

This is a clinical retrospective study of medical records of one patient who underwent surgical treatment for odontogenic myxoma.

Moreover, a review of the medical literature in PubMed and Scopus database was performed, covering articles published in Portuguese or English and using the following keywords (MeSH): “myxoma, odontogenic myxoma”.

The medical records of the subjects were used in this review and all the parameters of the surgical procedure and related devices were detailed.

### 2.1. Ethics

The institutional review board approved this study, and all subjects gave written informed consent.

## 3. Case Report

**Figure 1:**
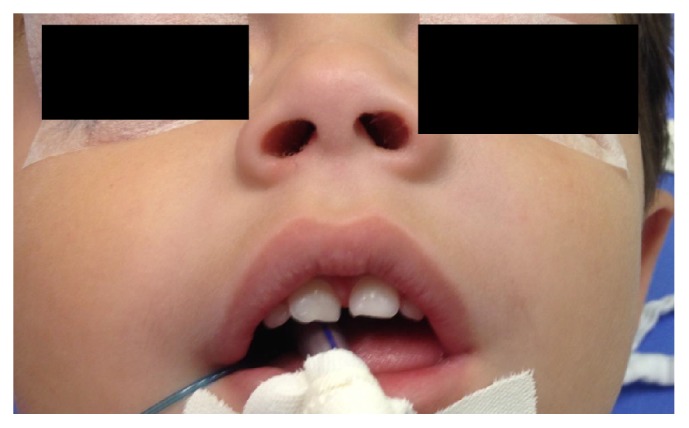
Face asymmetry before surgery.

A male patient, 2 years and 5 months old, presented at the otorhinolaryngology outpatient clinic with parents complaining of an enlargement and hardening of left maxilla region for 2 weeks (Figure 1). The parents denied a history of fever, weight loss, pain, secretion, surgery, dental procedures, or trauma. Physical examination showed a 2.5 cm lesion with fibroelastic characteristics and no signs of infection. Oroscopy, otoscopy, and neck examination showed no alterations.

**Figure 2:**
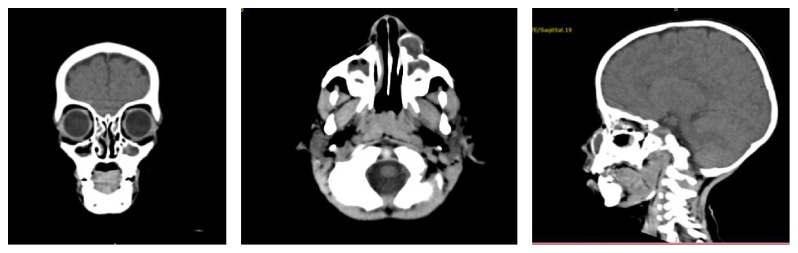
Coronal axial and sagittal CT: odontogenic myxoma left maxilla.

**Figure 3:**
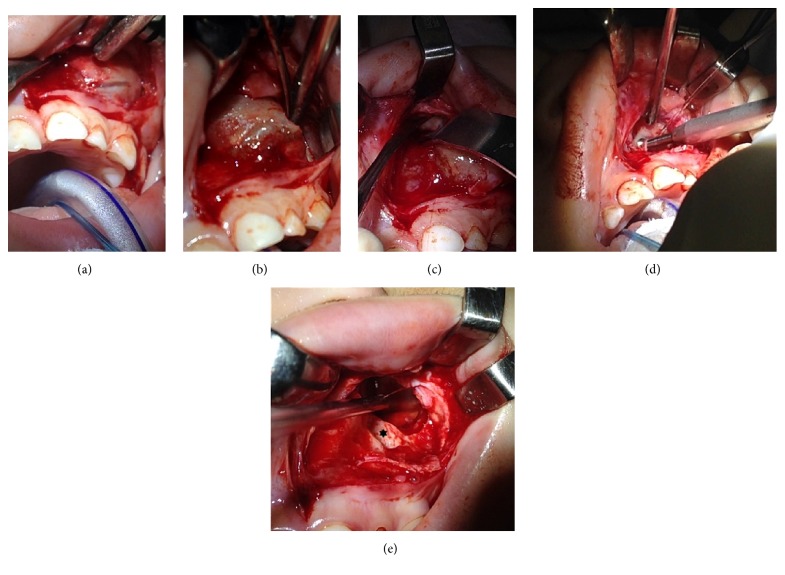
Surgery steps 2 to 5. ∗Dental germ removal.

A face tomography was completed and revealed a maxillary sinus cyst with odontogenic origin and associated bone erosion (Figure 2). Therefore, the main diagnosis hypothesis was odontogenic cyst, and the patient was submitted to surgery (Figure 3).

Access was obtained through a sublabial incision at anterior left maxilla wall, and a dissection along anatomical planes through the cyst wall covered by a bone capsule was performed (Figure 3(a)). Thereafter, dissection along the cyst limits and identification of bone erosion were performed (Figure 3(b)). Peripheral osteotomies in all the surrounding bone that was in direct contact with the lesion were made, and this bone was removed with a cutter (Figures 3(c) and 3(d)). Additionally, resection of the cyst through the opening of the bone capsule was followed by removal of the dental germs. The cyst had a gelatinous mass characteristic (Figure 3(e)).

The patient was discharged from hospital at the first day after surgery with no complaints and with antibiotics and local care (cleaning of the incision site and ice compresses).

**Figure 4:**
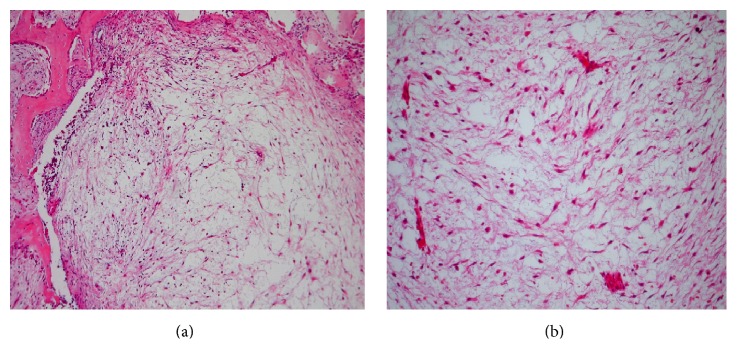
Odontogenic myxoma: (a) H&E ×10: a lobular and loose myxomatous tumor surrounded by alveolar bone. (b) H&E ×20: note the spindle and stellate cells in a myxoid stroma with few collagen fibrils and delicate capillary vasculature.

**Figure 5:**
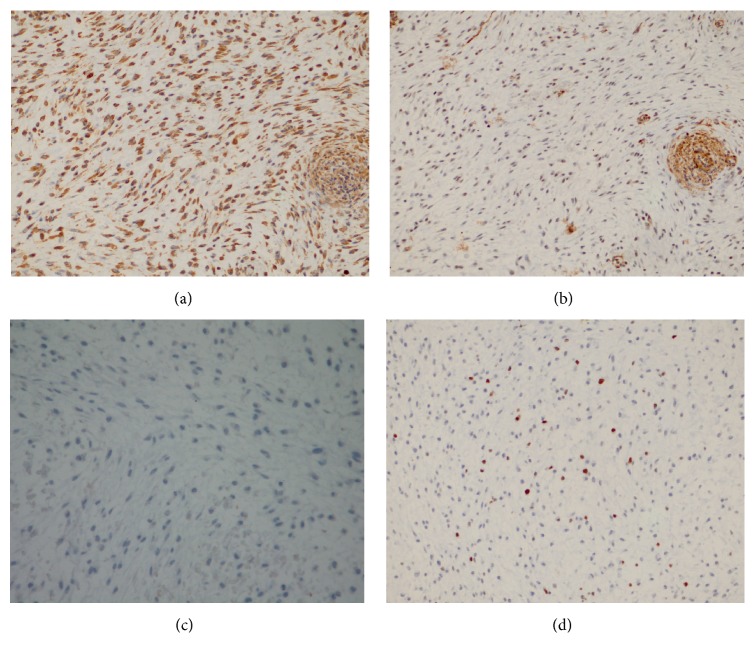
Immunohistochemical analysis: (a) diffuse positivity for vimentin, (b) focal expression for 1A4 auto control: vessels wall, (c) AE1-AE3 negative, and (d) Ki-67 expression around 5–8% of the nuclei.

The histology showed a lesion with an ill lobular appearance, surrounded by alveolar bone tissue. It is compounded by a cytologically bland spindled and stellated-shaped cells proliferation in an abundant, loose myxoid stroma that contains few collagen fibrils and delicate vessels (Figure 4). An immunohistochemistry analysis showed a diffuse expression against vimentin antibody, with focal reactivity for muscle-specific actin (Table 1 and Figure 5), favoring fibroblastic proliferation with some myofibroblastic differentiation.

**Figure 6:**
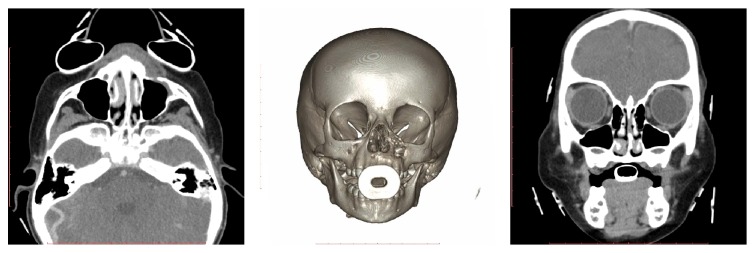
Coronal, axial, and 3D bone reconstruction showing no recurrence of the maxillary lesion.

Two months after the surgery, the patient's parents denied complaints, and a new tomography was requested, which showed no evidence of recurrence (Figure 6).

## 4. Discussion

According to the World Health Organization (WHO), odontogenic myxoma is an intraosseous benign but infiltrative neoplasm, classified under odontogenic tumors derived from mesenchyme and/or odontogenic ectomesenchyme with or without odontogenic epithelium. Histopathological features include spindle-shaped and round cells in an abundant loose mucoid/myxoid stroma with few collagen fibrils. Capillaries, odontogenic epithelium, mast cells, plasmacytoid, and residual bony trabeculae can also be found [4]. Immunohistochemistry findings reveal vimentin expression but not cytokeratin, desmin, or neurofilaments [5]. This tumor can also present as a capsule, which can be associated with the locally aggressive nature of the neoplasm. Odontogenic tumors are a rare entity in children. Fang et al. presented a series of 310 cases of odontogenic tumors or tumor-like lesions in which four patients had myxoma, and all patients underwent aggressive surgery, with no recurrence observed [6]. Recurrence rates for myxomas in the literature vary from 5 to 10%: however, there is no consensus information in the literature [7].

Due to its slow growing characteristic, patients typically do not have many symptoms, and an investigation with CT and MRI is essential both for surgical planning and for differential diagnosis with lesions that could contraindicate biopsy [5]. A wide resection with 10 to 15 mm margins is recommended because of the high recurrence rates. Nevertheless, there is no guideline concerning surgical approach in these patients, and some authors note that enucleation and curettage can be effective and less aggressive, considering large resections along with bone grafting and rehabilitation being necessary [1, 2, 5, 8]. Boffano et al. suggested conservative surgery by enucleation and curettage when lesions were smaller than 3 cm, whereas a segmental resection with immediate reconstruction is favored in patients with larger lesions [9].

Kleiber et al. presented a very aggressive case of myxoma with both mandible and maxillae commitment in a three-year-old patient who underwent two surgical procedures and previous chemotherapy. After a large resection, this patient did not experience disease recurrence over a 4-year follow-up period [10]. Kiresur and Hemavathy also performed an extended resection of the jaw in a 17-year-old patient because resection procedures minimize the risk of involvement of vital structures and reduce the recurrence rate [11]. Limdiwala and Shah described two odontogenic myxomas of the maxillae, both in adults that underwent conservative surgery, but follow-up was not described in this paper [12].

Kawase-Koga et al. presented a literature review of 44 cases with patients (50% male) presenting a median age of 31.9 years. Forty-five percent underwent conservative surgery, and, in this group, a 15% recurrence rate was observed. The other patients underwent radical procedures, and, in this group, no recurrence was presented. The follow-up was a median length of 46 months. Within those cases, three cases were children that were 6, 7, or 12 years old; one of those children underwent radical surgery, but none of the children presented with recurrence [2]. Kadlub et al. presented a review of 20 cases of infants (under two years old) with odontogenic myxoma. These patients had a median age of 14,9 months, and all cases had maxilla commitment; 62,5% of the cases had conservative tumor resection, of which one presented recurrence. The median follow-up was 2,84 months [1]. This patient was subjected to peripheral osteotomies, which is a compromise between aggressive bone resection and conservative cyst extraction or curettage. Although there is only a one-year follow-up period, this technique showed great effectiveness.

Based on histopathologic features a myxoma may be confused with other myxoid neoplasms of the jaw, such as the rare chondromyxoid fibroma or myxoid neurofibroma; the first could have areas of cartilaginous differentiation, whereas myxoid neurofibromas tend to have scattered lesional cells, similar to myxoma, but both of these tumors are positive for S-100 protein, which is not observed on odontogenic myxoma [13]. Also the bland cellularity of the myxoma and the negativity for desmin and Myogenin speak against a botryoid-type embryonal (myxoid) rhabdomyosarcoma, of the head and neck in children. Myxoid change in an enlarged dental follicle or the dental papilla of a developing tooth may be microscopically similar to a myxoma, but the clinical, radiographic features and all the pathologic and immunohistochemical findings observed, in this case, favor the diagnosis of an odontogenic myxoma.

There is a large spectrum of differential diagnosis of odontogenic raging from benign to malignant diseases, in both children and adults. Especially concerning children, the diagnosis prior to surgery is essential because a functional and esthetic defect can be more complex, even considering a recurrence rate [7]. Consequently, a follow-up is extremely necessary in the two years after surgery, when recurrences occur more often, but it is also recommended that these patients never get discharged from the outpatient clinic [2].

## 5. Conclusion

Odontogenic myxoma at pediatric ages is a rare pathology; therefore, few studies have been performed in this field. Differential diagnosis is essential in children, and CT and MRI have important roles in this process.

The evidence supports conservative resections in these patients due to the higher morbidity of aggressive surgeries. This paper also presents a middle ground between aggressive and conservative surgery by peripheral osteotomies. There is a need for evidence-based recommendation in terms of treatment of those cases. Especially in infants, long-term follow-up is important due to the recurrent nature of this disease and the long-term life expectancy in these patients.

## Figures and Tables

**Table 1:** Immunohistochemical panel.

Vimentin	Diffusely positive
CD34	Negative (positive control: vessel)
AE1/AE3	Negative (positive control: epithelial component of local developing teeth germs)
Desmin	Negative
S-100	Negative
1A4	Focus of positivity
Myogenin	Negative
Ki67	5–8% of the cells
